# Research Progress for RNA Modifications in Physiological and Pathological Angiogenesis

**DOI:** 10.3389/fgene.2022.952667

**Published:** 2022-07-22

**Authors:** Hui-Ming Chen, Hang Li, Meng-Xian Lin, Wei-Jie Fan, Yi Zhang, Yan-Ting Lin, Shu-Xiang Wu

**Affiliations:** ^1^ Key Laboratory of Gastrointestinal Cancer (Fujian Medical University), Ministry of Education, School of Basic Medical Sciences, Fujian Medical University, Fuzhou, China; ^2^ Fujian Key Laboratory of Tumor Microbiology, Department of Medical Microbiology, Fujian Medical University, Fuzhou, China

**Keywords:** RNA modifications, angiogenesis, tumor, m^6^A, m^5^C

## Abstract

As a critical layer of epigenetics, RNA modifications demonstrate various molecular functions and participate in numerous biological processes. RNA modifications have been shown to be essential for embryogenesis and stem cell fate. As high-throughput sequencing and antibody technologies advanced by leaps and bounds, the association of RNA modifications with multiple human diseases sparked research enthusiasm; in addition, aberrant RNA modification leads to tumor angiogenesis by regulating angiogenesis-related factors. This review collected recent cutting-edge studies focused on RNA modifications (N^6^-methyladenosine (m^6^A), N^5^-methylcytosine (m^5^C), N^7^-methylguanosine (m^7^G), N^1^-methyladenosine (m^1^A), and pseudopuridine (Ψ)), and their related regulators in tumor angiogenesis to emphasize the role and impact of RNA modifications.

## Introduction

The epitranscriptome has been thought of as an important layer for the regulation of gene expression, which was expected to become a key target in the treatment of cancers (Dong and Cui, 2020). Also, currently, over 180 different types of chemical modifications on ribonucleic acid (Boccaletto et al., 2022) have been discovered in all three life domains and viruses (Krug et al., 1976; Canaani et al., 1979; Deng et al., 2015) that widespread exist on several types of RNA, including small nucleolar RNA (snoRNA), noncoding RNA (ncRNA), ribosomal RNA (rRNA), transfer RNA (tRNA), and messenger RNA (mRNA). According to the incidence of cancer rising in the past few decades, the associations between RNA modifications with tumor development were widely studied to try find a new therapeutic target (Zhao et al., 2017a). However, the quantitative methods to detect RNA epigenetics with highly accuracy and sensitivity are still under development, and the function and mechanism of these chemical modifications on different types of RNAs is under exploration.

**FIGURE 1:**
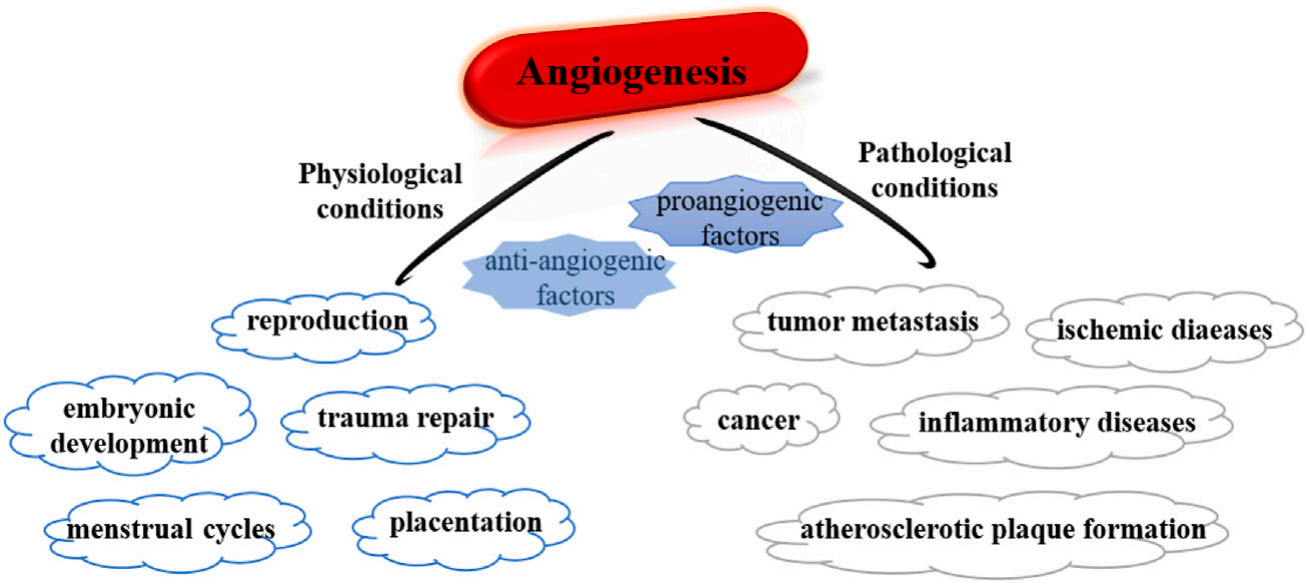
Summary of roles of angiogenesis under physiological and pathological conditions.

In recent decades, microRNAs (miRNAs) have been considered one of the factors of angiogenesis, affecting the processes associated with neovascularization that include angiogenesis and arteriogenesis (Weber, 2013; Welten et al., 2016). In the angiogenesis, the new capillaries will growth from the existing vasculature and postcapillary venules and then redistribute local blood flow toward ischemic areas, which contributes to relieving ischemia (van der Kwast et al., 2019). Under physiological conditions, angiogenesis is strictly regulated by various cytokines and is crucial to embryonic development, trauma repair, reproduction, and menstrual cycles (Ma et al., 2020), while pathological angiogenesis is a continuously uncontrolled process that leads to various diseases (Jeong et al., 2021) (Figure 1). Under hypoxia, vascular endothelial growth factor (VEGF) is a mitogen that directly stimulates endothelial cells (ECs) division and proliferation, thereby resulting in new capillaries. Apart from ECs, vascular smooth muscle cells, fibroblasts, and immune cells also play vital parts by affecting and regulating ECs function as well as secreting angiogenic factors (Schwartz and Mitchell, 1962; Noonan et al., 2008; Newman et al., 2011). Furthermore, direct angiogenic factors are highly correlated and are jointly involved in the formation of vascular networks in tumors (Viallard and Larrivée, 2017). In addition, angiogenesis-related factors interact and influence each other, for example, VEGF stimulates ECs to secrete fibroblast growth factor (FGF), which in turn activates the VEGF system for angiogenesis (Hosaka et al., 2020). The most widely studied VEGF family, especially VEGFA, is a key angiogenic factor regulating ischemic diseases. VEGFA expression was influenced by histone methylation, ncRNA, and other epigenetic regulatory networks. Lack of VEGF leads to embryonic body death, while overexpression of VEGF promotes tumor angiogenesis (Melincovici et al., 2018). VEGF was overexpressed in liver tissue dysplasia and premalignant cirrhosis stage, suggesting that VEGF is bound up with the grade of hepatocellular carcinoma (HCC) (Hamdy et al., 2020). In renal cancer tissues of different clinical stages, aberrant expression of VEGF may be correlated with the deterioration of disease (Hirsch et al., 2020). Besides, overexpression of VEGF is associated with clinicopathological stage in patients and is expected to be a major factor in the prediction of gastric cancer recurrence (Pang et al., 2018).

Because of the dynamics of RNA modifications, cells respond to external signals accurately and quickly. The ability to adapt to microenvironmental changes, such as stress and hypoxia, is a factor for tumor cell survival, which indicates that RNA modifications may play significant part in tumors. Angiogenesis is a major feature of tumors and mainly formed by genetic mutation or epigenetic alterations (Hanahan and Weinberg, 2011). Researchers showed that RNA modifications also participate in certain pathological processes, affecting gene transcription and chromatin integrity and even regulating signaling pathways of the cell cycle, proliferation, differentiation, apoptosis, and tumorigenesis (Nombela et al., 2021). Of note, substantial evidence indicates that abnormal expression of RNA modification-related regulators comprises of methyltransferases, demethylases, and binding proteins (Pan et al., 2018), which are associated with angiogenesis and tumorigenesis (Blanco et al., 2014a; Barbieri and Kouzarides, 2020a). Other RNA modifications participated in multiple biological processes, which have been proven in recent studies. For instance, 2ʹ-O methylation, which is hypermodified in rRNA, reducing efficient translation and affecting growth and sensitivity to antibiotics (Liang et al., 2009). m^5^C is commonly found in tRNA and rRNA and is associated with protein translation (Blanco et al., 2014a; Schosserer et al., 2015; Janin et al., 2019a). Next, pseudoreuridine, in yeast mRNA expression, increases under heat shock and starvation conditions (Carlile et al., 2014). m^1^A is primarily enriched within 5ʹ UTR, and located upstream vicinity of start codons contributes to the structural stability of RNA, which is associated with increased translation and induction of stress (Dominissini et al., 2016a).

**FIGURE 2:**
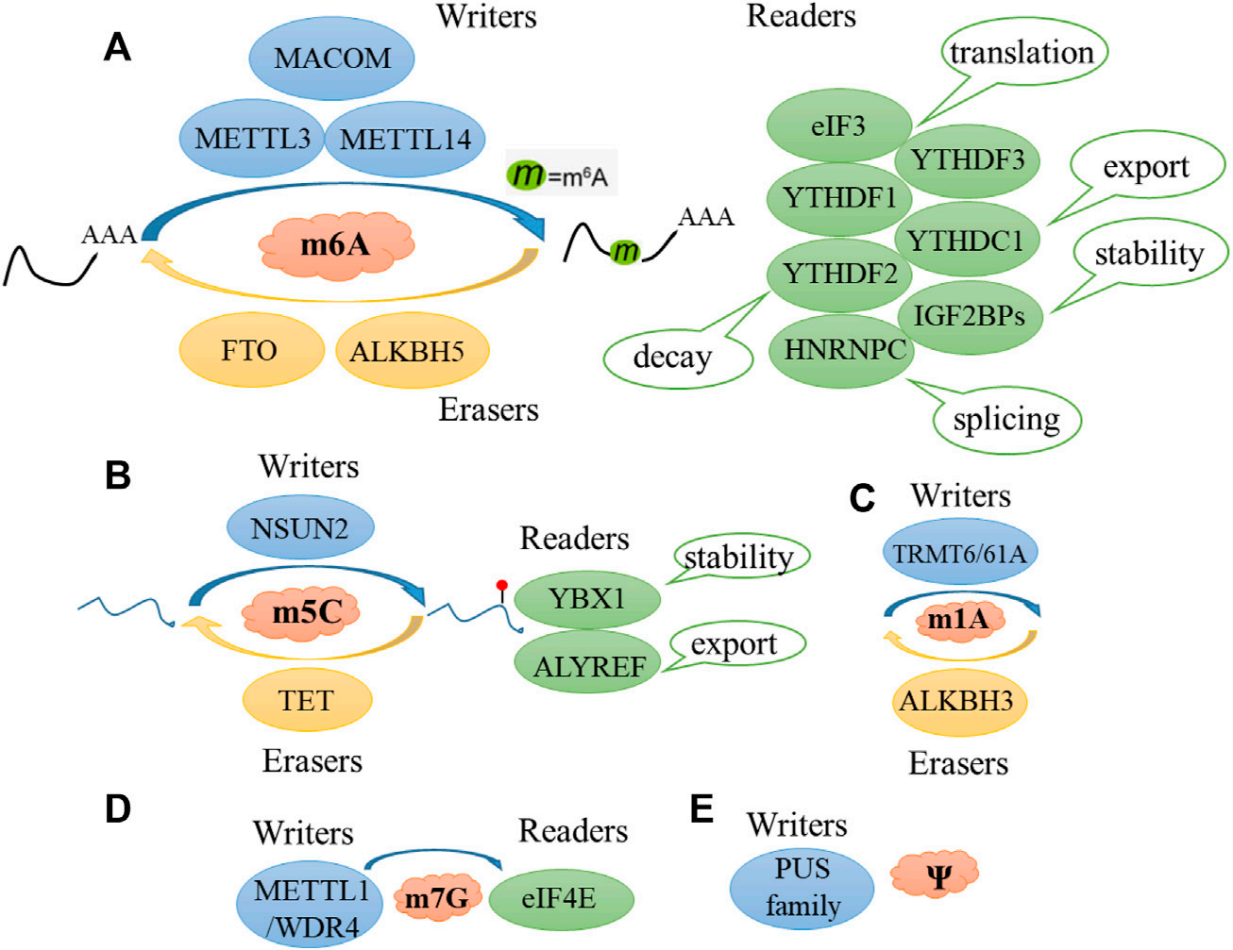
Major classes of RNA modification. Schematic diagram of m^6^A **(A)**, m^5^C **(B)**, m^1^A **(C)**, m^7^G **(D)**, Ψ **(E)**, and regulated by methyltransferase (writers), demethylases (erasers), and some specific proteins (readers).

Considering that angiogenesis is an important physiological and pathological process, RNA modification is the research front in epigenetics. Thus, in this review, we systematically summarized the RNA modification-associated angiogenesis pathways, including m^6^A, m^5^C, m^7^G, m^1^A, and Ψ (Figure 2).

## RNA Modification Type

### RNA m^6^A Methylation

In 1974, N^6^-methyladenosine (m^6^A) was identified in human mRNA for the first time (Desrosiers et al., 1974). Also, previously, some methods have been developed to detect m^6^A modification, including dot blots (Wang et al., 2018), electrochemical immunosensor method (Yin et al., 2017), RNA photo-crosslinkers, and quantitative proteomics (Arguello et al., 2017); however, they cannot determine the precise location of m^6^A. The LC/MS analysis suggested that m^6^A is the most prevalent mRNA modification in mammals. With the advent of MeRIP-Seq (Meyer et al., 2012)/m^6^A-seq (Dominissini et al., 2012), the m^6^A conserved motif RRACH was discovered, where the most classical motif is GGACU (R = Guanine, Adenine, and H = Adenine, Cytosine, Uracil); meanwhile, miCLIP technology enabled correct identification of m^6^A residues at base-resolution (Linder et al., 2015). Further, at least 12 methods were developed to detect methylation based on the NGS platform (Wang and Jia, 2020). Right now, at least 178,049 human modification sites have been reported based on the high-resolution sequencing techniques (Tang et al., 2021a). Moreover, the evolutionary conservation of m^6^A was discussed among mammals (Dominissini et al., 2012), primates (Ma et al., 2017), and plants (Miao et al., 2021), reflecting the potential correlation between m^6^A richness with gene-specific structure and function (Song et al., 2021a).

RNA m^6^A modification is a reversible mark. The methyl group from S-adenosylmethionine can be added on the adenine by methyltransferase (writers), and the methyl group can be removed from the methylation sites by demethylases (erasers). Next, some specific proteins (readers) will recognize m^6^A that affects these regulatory mechanisms and exert important biological effects. Furthermore, m^6^A affects RNA metabolism in different stages, such as mRNA translation (Frye and Blanco, 2016), splicing (Liu et al., 2017), degradation (Shima et al., 2017), secondary structure (Kierzek et al., 2022), and stability (Wang et al., 2014). In general, m^6^A plays crucial roles of RNA processing in both the nucleus and cytoplasm (Zhao et al., 2017b).

### m^6^A Writers

In eukaryotic cells, m^6^A methyltransferases are composed of three components: a main catalyze domain methyltransferase-like 3 (METTL3), a substrate recognition domain methyltransferase-like 14 (METTL14), and the m^6^A-METTL-associated complex (MACOM). METTL3 exhibits the S-adenosine methionine (SAM)-binding motif, and METTL14 demonstrates a methyltransferase domain also, which shares 22 percentage amino acids sequences with METTL3 (Śledź and Jinek, 2016). Next, MACOM comprises four components: RBM15, Zc3h13, WTAP, and VIRMA (He et al., 2019). RBM15 can recognize target mRNA and recruit the other components of the methyltransferase complex: WT1 associated protein (WTAP). WTAP not only acts as a splicing factor regulating the methylation and location of the heterodimer (Ping et al., 2014) but also regulates complex formation. Next, Zc3h13 recruits protein that can connect the other two adaptors, RBM15 and WTAP (Knuckles et al., 2018). In recent years, another active single methyltransferase, METTL16, was found to maintain SAM homeostasis, which can add a methyl group on the SAM synthase transcript, which contributes to the methylation of a small nucleolar (snoRNA) (Mendel et al., 2018).

### m^6^A Erasers

RNA demethylases were not discovered until 2011. Jia and her colleagues (Jia et al., 2011a) proved the m^6^A is the substrate of fat mass and obesity-related proteins (FTO), and the FTO will induce the demethylation of m^6^A. FTO can oxidize m^6^A in an indirect way, which will generate two intermediates: hm^6^A and f^6^A (Jia et al., 2011b). ALKBH5 is another eraser, which is homolog of FTO (Zheng et al., 2013) and catalyzes the direct removal of m^6^A methylation, acting on mRNA preferentially (Zhou et al., 2020).

### m^6^A Readers

Readers serve as the recognizer of m^6^A methylation that recruits complexes or cytokines to bind with RNA to execute different functions. Family proteins (YTHDF1-3 & YTHDC1-2) containing the YTH domain (Li et al., 2014) are one group of m^6^A readers. YTHDF1, in synergy with YTHDF3, facilitates translation and promotes mRNA binding to the ribosome. Next, YTHDF2 is the first recognized m^6^A reader, whose main substrate is the m^6^A on 5ʹ UTR, and it competes for ribosomes mediating mRNA translation (Du et al., 2016). YTHDC1 is primarily present in the nucleus to regulate gene splice exon selection, and YTHDC2 mediates mRNA degradation and regulates translation in a m^6^A-dependent manner (Kretschmer et al., 2018; Chen and Wong, 2020). Next, insulin-like growth factors (IGF2BPs) is another group of m^6^A readers that stabilize mRNA based on m^6^A-dependent manner (Huang et al., 2018). Also, eIF3 (Meyer et al., 2015) and other “indirect” readers recognize m^6^A to exert specific functions (Wu et al., 2019a). The aberrant expression of these m^6^A-associated downstream proteins were observed in tumorigenesis, angiogenesis, and immunoregulation.

### Roles of m^6^A in Tumors

m^6^A methylation participating in the process of tumorigenesis was proved by many studies, including proliferation (Liu et al., 2018), invasion (Yue et al., 2019), and immune system evasion (Han et al., 2019). Based on the potential samples with lung and colon cancer, the METTL3 is considered as an oncogene to facilitate the development of the disease (Lin et al., 2016). Researchers reported that upregulated FTO enhanced the development and metastasis of breast cancer cell through downregulation of BCL2 interacting protein 3 (BNIP3) expression in both vitro and vivo experiment (Niu et al., 2019). Also, overexpression of YTHDC2 is observed in colon cancer, which is considered as a mark associated with poor prognosis (Tanabe et al., 2016).

### RNA m^5^C Methylation

5-methylcytosine (m^5^C) is another common and dynamic RNA marker found in most organisms. Based on biochemical studies, the m^5^C methylated tRNA and rRNA were observed demonstrating various molecular functions (Blanco et al., 2014b; Janin et al., 2019a). For example, m^5^C alters the rRNA conformation to regulate synthesis of ribosome, thereby affecting translation fidelity. The tertiary structures of tRNA are maintained by m^5^C, which is evolutionarily conserved. Also, recent high-throughput studies based on bisulfite treatment or immunoprecipitation techniques proved that m^5^C modification also appears on mRNA (Huang et al., 2019).

Similar to this, m^5^C is also a reversible process. The m^5^C methylation can be added on RNAs by different enzymes, including the NSUNs family and DNMT2 (Cui et al., 2017). The ten-eleven translocator family is most likely to be a m^5^C eraser catalyzing the demethylation of m^5^C, but the mechanism of action remains to be demonstrated (Fu et al., 2014). In recent studies, the Aly/REF export factor (ALYREF) (Yang et al., 2017) was proven to demonstrate an ability to recognize m^5^C sites and can be considered as a m^5^C reader, which facilitates m^5^C-dependent export; furthermore, the same research group suggested that the m^5^C sites are substrates of the YB protein family YBX1 (Chen et al. 2019c) and YBX2, which can help mRNA stabilization and liquid-liquid phase separation in m^5^C-dependent manner, respectively.

### Role of m^5^C in Tumors

Also, m^5^C is involved in tumor formation and various diseases. One study suggested that overexpression of NSUN2 may contribute to the proliferation of mouse fibroblasts (Perlaky et al., 1992). Meanwhile, NSUN2 is upregulated in lung cancer (Saijo et al., 2001), prostate cancer (Bantis et al., 2004), and breast carcinoma (Freeman et al., 1991), and it is used as a marker of poor prognosis. Also, to reduce the stability of p57 mRNA in gastric cancer, the NSUN2 is responsible for the m^5^C methylated sites on the 3’ -untranslated regions of p57, thus suppressing the expression of p57, which may lead to the development of gastric cancer (Mei et al., 2020). Furthermore, the overexpression of NSUN4 is strongly related to HCC (He et al., 2020). Also, downregulation of NSUN5 is observed in glioblastoma patients and is related to low survival (Janin et al., 2019b).

### RNA m^7^G Methylation

N^7^-methylguanosine (m^7^G) is a commonly observed in positively charged methylated nucleotides under physiological conditions (Agris, 1996a) and was a constituent of mRNA cap structures (Ramanathan et al., 2016). Meanwhile, it is conserved between eukaryotes and archaea. As the part of 5′cap structure, m^7^G methylation participates in the mediation of multiple biological processes, such as pre-mRNA splicing, transcription termination, exosomal degradation, and mRNA export (Chen and Guo, 2016). Next, m^7^G is catalyzed by the METTL1/WDR4 complex and is recognized by eIF4E in humans, which affects the translation efficiency of transcripts at the m^7^G site (Lin et al., 2018).

### RNA m^1^A Methylation

N^1^-methyladenosine (m^1^A) is a reversible modification with a high content in tRNA but a relatively low abundance in mRNA (Dominissini et al., 2016b; Shima and Igarashi, 2020). Also, m^1^A is a common positive electrostatic methylated modification under physiological conditions that produce electrochemical effects and is important for the biological functions and secondary structure of tRNA (Agris, 1996b). Next, m^1^A methylation in human mRNA is a rare modification that blocks Watson-Crick base pairing and alters the mRNA structure and initiation of translation (Li et al., 2016; Safra et al., 2017). Scientists use this feature to create high-throughput m^1^A sequencing technology (Hauenschild et al., 2015) to identify hundreds of m^1^A sites on mRNA (Li et al., 2017).

Next, currently, the TRMT6/61A complex is the only known methyltransferase which is responsible for mRNA m1A methylation (Dominissini et al., 2016b), which is demethylated by the AlkB homolog protein ALKBH3 (Woo and Chambers, 2019). In mitochondria, TRMT61B and TRMT10C methylate m^1^A sites in mt-tRNA transcripts (Chujo and Suzuki, 2012; Vilardo et al., 2012). In addition, m^1^A is also found in human and mouse 28S rRNAs, which is methylated by RRP8 (Waku et al., 2016).

Of note, ALKBH3 can demethylate m^1^A tRNA to promote protein synthesis, which may contribute to the cancer development (Ueda et al., 2017). Next, ALKBH1 demethylates other targeted tRNAs, affecting translation by reducing the synthesis of tRNAs in proteins (Liu et al., 2016).

### Pseudouridine

Pseudouridine (Ψ), often termed as the fifth-class nucleotide, an isomerization of uracil, is the first identified posttranscriptional modification (Davis and Allen, 1957). Based on the Davis and Allen’s research, at least five percentage ribonucleic acid in transcriptome were identified as pseudouridine, and Ψ is considered as the most abundant RNA modification (Hamma and Ferré-D'Amaré, 2006). An important difference from methylation, pseuduridine is considered as an irreversible modification (Nielsen and Arison, 1989). Also, the reader of pseuduridine is still unknown.

Pseudouridylation plays a different role depending on the RNA type that is modified, and the presence of Ψ is crucial to the cancer. Dyskerin pseudouridine synthase 1 (DKC1), the enzyme catalyzing pseudouridylation, proved that it is disease associated. For example, its mutation will result in a loss of pseudouridines in mature rRNA, increase the risk to suffer the X-linked dyskeratosis congenita (X-DC), and fail the differentiation of hematopoietic stem cells to increase the risk of cancer (Kirwan and Dokal, 2008). In 28S rRNA, a reduction in pseudouridylation leads to the dysregulation of the critical mRNA translation of VEGF and p53 (Barbieri and Kouzarides, 2020b). In tRNA, aberrant expression of snoRNA24-guided Ψ influenced translation efficiency and increased stop codon readthrough frequencies, thereby driving the development of HCC (McMahon et al., 2019). Thus, pseudouridine will be a potential biomarker and is expected to be a potential anticancer target from a clinical perspective.

## Role of RNA Modification in Angiogenesis

### m^6^A in Physiological Angiogenesis

RNA m^6^A modification, as an important regulator of mRNA biology, often occurs in many physiological processes and plays a pivotal role in ontogeny (Liu et al., 2019). Also, currently, METTL3-mediated m^6^A methylation is vital in responding to hypoxic stress and promoting angiogenesis. *In vitro* experiments revealed that METTL3 participates in the regulation of endothelial cell viability, proliferation, and migration. Next, mechanistically, METTL3 methylates LRP6 and disheveled 1 (DVL1) for m^6^A to regulate Wnt signaling and further exert its angiogenic role (Yao et al., 2020). In osteogenesis, the high expression of METTL3 will activate the PI3K/AKT signaling pathway of endothelial progenitor cells to enhance the cell growth, migration ability, tube formation activity, and ultimately promoting EPCs angiogenesis (Jiang et al., 2021). Bone marrow mesenchymal stem cells (BMSCs) can secrete VEGF to promote local angiogenesis, while METTL3 is highly expressed in osteogenic differentiated BMSCs, and the knocking down of METTL3 leads to the lower expression of VEGFA (Tian et al., 2019). Another *in vivo* experiment showed that silencing the eraser FTO in ECs can induce hyper-methylation on critical proangiogenic genes, such as FAK, which were recognized by the m^6^A reader YTHDF2 to induce RNA decay, thus regulating ocular angiogenesis (Shan et al., 2020). In comparison, angiogenesis is regulated by miR-4729, which inhibits METTL14 to decrease the m^6^A methylation of TIE1 and further inhibit TIE1/VEGFA signaling pathways in vascular ECs (Liu et al., 2021). In addition, FTO plays a vital role in cardiac contraction function, remodeling, regenerative repair, and cardiac homeostasis. Under ischemic conditions, overexpression of FTO reduced the abnormal modification level of m^6^A in the whole heart and improved the expression of related proteins, thus reducing myocardial fibrosis and enhancing angiogenesis in ischemic myocardium (Mathiyalagan et al., 2019).

### m^6^A in Pathological Angiogenesis

Vascular vessels are important channels of tumor metastasis, and angiogenesis is a hallmark of tumor aggressiveness (Goel et al., 2011). The progression of tumor angiogenesis is mutually controlled by a range of stimulators and suppressors. Also, current evidence exists that m^6^A affects tumor angiogenesis (Han et al., 2021).

In multiple types of cancer, overexpression of METTL3 promoted angiogenesis by enhancing oncogenes (Wang et al., 2020a). Also, the study found that METTL3 targets EphA2 and VEGFA through a IGF2BP3 dependent mechanism to promote the formation of vasculogenic mimicry in the colorectal cancer (CRC) activated PI3K/AKT and ERK1/2 signal pathway, thus promoting the development of tumors (Liu et al., 2022a). Also, METTL3 upregulated expression of JAK2 and then activated JAK2/STAT3 pathway in ox-LDL-induced human umbilical vein endothelial cells (HUVECs), and IGF2BP1 directly binds with JAK2 RNA on a m6A site, which promoted angiogenesis (Dong et al., 2021). Furthermore, a study also suggested that overexpression of METTL3 facilitated gastric cancer liver metastasis and angiogenesis *in vivo*, which correlated with methylation states of secreted heparin-binding growth factor (HDGF). Also, HDGF is the substrate of and the IGF2BP3, which stabilized HDGF mRNA and subsequently caused tumor angiogenesis (Wang et al., 2020a). Next, METTL3 can facilitate the maturity of miR-143-3p by splicing the precursor. Further, miR-143-3p can be a target to the vasohibin-1 (VASH1) to inhibit its translation, which leads to the hypoubiquitylation of VEGFA to inhibit its degradation and promotes angiogenesis in lung cancer (Wang et al., 2019). In bladder cancer tissues, TEK/PI3K/VEGF cascades were also enhanced by METTL3, which is involved in angiogenesis in tumor cells (Han et al., 2021). In recent studies, m^6^A methylation existing on circular RNAs (circRNAs) was proved, which contributes to the angiogenesis of CRC (Chen et al., 2020a; Chen et al., 2020b). One study found that ALKBH5 and YTHDF3 negatively regulated circ3823 and were significantly reduced in CRC. Circ3823 inhibited expression of miR-30c-5p to enhance the translation of TCF7. Further, the expression of MYC and CCND1 were induced by the TCF7 to facilitate CRC growth, metastasis, and angiogenesis (Guo et al., 2021). Furthermore, Chang et al. (2020) reported that the overexpressed YTHDF3 is observed in the clinical patients with brain metastases. The overexpressed YTHDF3 may facilitate the cancer cell to go through the blood-brain barrier because of its enhancement of the translation of several brain metastasis-associated m^6^A-enriched transcripts, namely, ST6GALNAC5, GJA1, and EGFR.

Also, gene ontology results suggested that FTO participates the regulation of angiogenesis. Next, low expression of FTO is correlated with microvessel density (MVD) in intrahepatic cholangiocarcinoma (ICC) (Rong et al., 2019), which is predicted to be associated with poor prognosis.

Moreover, studies showed that overexpression of YTHDF2 suppresses tumor growth in hepatocellular carcinoma. In terms of the relationship between YTHDF2 and tumor vessels, a study suggested that serine-type endopeptidase inhibitor 2 (SERPINE2) was upregulated in YTHDF2-silenced cells, thereby promoting angiogenesis and growth of HCC (Hou et al., 2019). Studies showed that microRNA-320b (miR-320b) can inhibit the proliferation of cancer cells and lead to apoptosis (Zhang et al., 2019). In lung cancer, the expression of IGF2BP2 was inhibited by overexpressing miR-320b. As the downstream of IGF2BP2, the stability of thymidine kinase 1 (TK1) mRNA is reduced subsequently because of the hypoexpressed IGF2BP2. Considering that the TK1 is important kinase in angiogenesis, the miR-320b suppressed cancer development by inhibiting IGF2BP2/TK1 manner (Ma et al., 2021). A recent report showed that IGF2BPs are associated with tumor progression and angiogenesis in colon cancer (Ye et al., 2016). Downregulated IGF2BP3 expression significantly reduced the m^6^A modification recognition of CCND1 and VEGF, which decreased both mRNA expression and stability. The m^6^A reader IGF2BP3 represses angiogenesis in colon cancer by regulating VEGF, thus inhibiting colon cancer angiogenesis (Yang et al., 2020). Downregulated WTAP expression in brain arteriovenous malformations (AVMs) and desmoplakin (DSP) mRNA is degraded rapidly because of the reduction in m^6^A methylation. In addition, the knockdown of WTAP will activate the Wilmsʹ tumor 1 (WT1) to degrade the β‐catenin, repressing the Wnt signaling pathway to suppress angiogenesis of ECs (Wang et al., 2020b).

**FIGURE 3:**
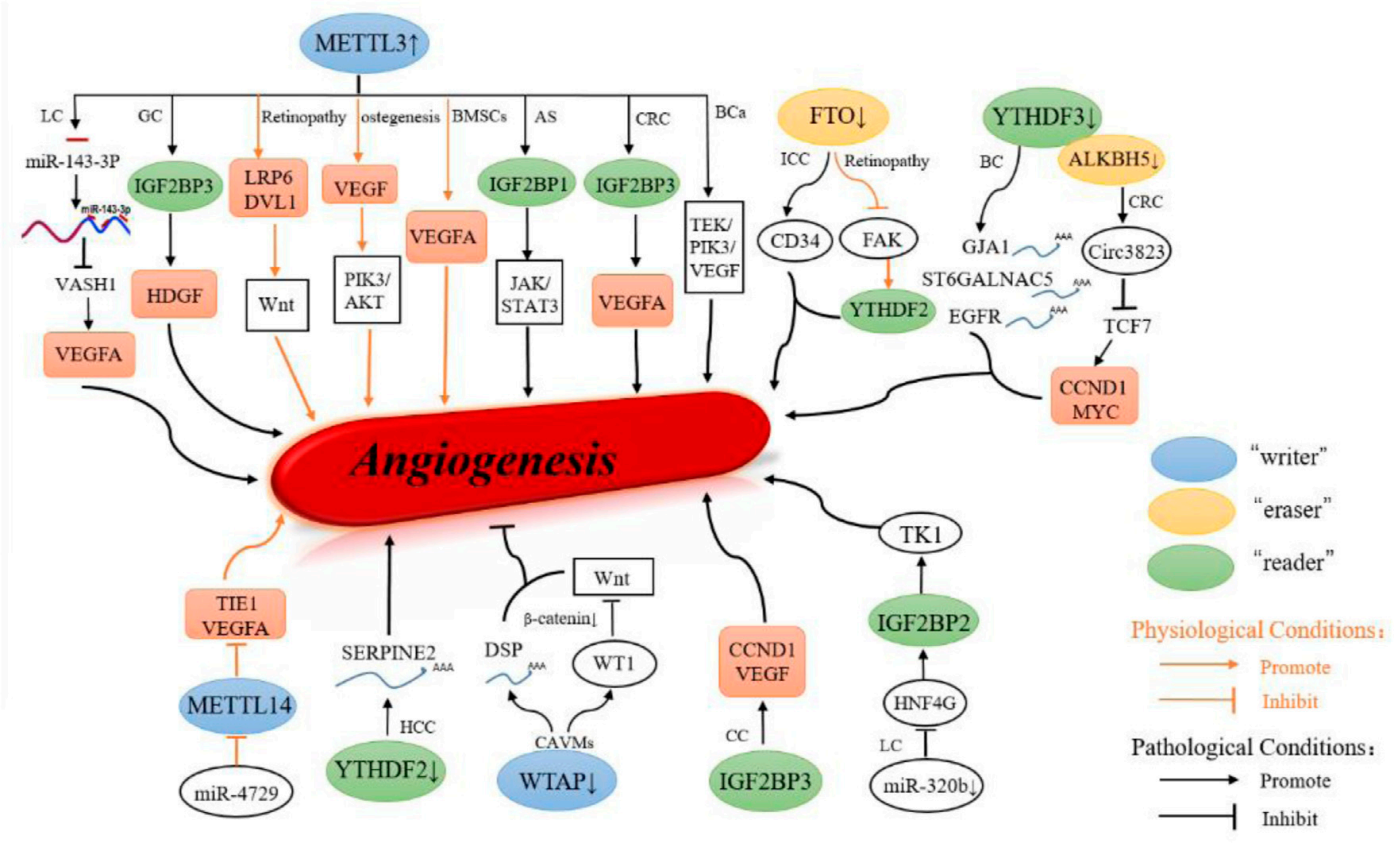
Role of m6A modification in angiogenesis. m6A is important to maintain the homeostasis of angiogenesis in physiological condition, and aberrant regulation of m^6^A regulators were observed in pathological condition.

In conclusion, N^6^-methyladenosine participates the regulation of angiogenesis under physiological or pathological conditions both (Figure 3).

### m^5^C in Angiogenesis

**FIGURE 4:**
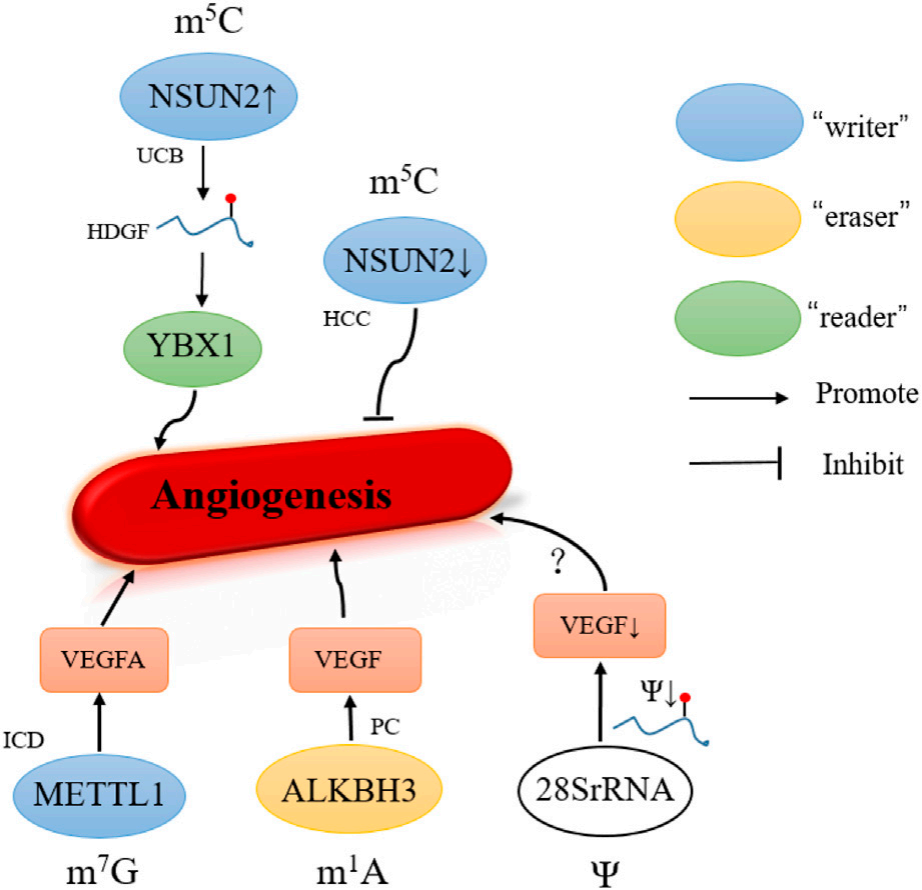
Role of other modifications in angiogenesis. UCB, urothelial carcinoma of the bladder; HCC, hepatocellular carcinoma; ICD, ischemic cardiovascular disease. PC, pancreatic cancer.

In human diseases, 5-hydroxymethylation has also been found to be altered in cancer and angiogenesis (Ma et al., 2022). In recent years, the m^5^C writer NSUN2 has been studied most extensively. Knockout of NSUN2 significantly reduced the number of invaded cells and cell cord formation on Matrigel, thereby inhibiting the invasion, metastasis, and angiogenesis of HCC (Sun et al., 2020). To promote the UCB development, overexpression of NSUN2 and YBX1 stabilized the HDGF mRNA by regulating the m^5^C methylation site on its 3′ untranslated region (Chen et al., 2019a) (Figure 4).

The molecular dynamics and function of RNA m^5^C modification are still in their infancy, and the role of m^5^C in angiogenesis and tumor progression is still poorly understood.

### m^7^G in Angiogenesis

METTL1 is a key gene involved in m^7^G methylation that demonstrates promising functions in angiogenesis regulation and could be a treatment target of vascular diseases (Deng et al., 2020). In HUVECs, to enhance the angiogenesis, METTL1 upregulates m^7^G methylation of mRNA in a complex ischemic environment. Also, the METTL1 methylated adenine of VEGFA to enhance its translation to promote postischemic angiogenesis (Zhao et al., 2021). As a consequence, the METTL1 could be considered as a potentially innovative therapeutic target in peripheral arterial disease (PAD) in clinical treatment.

### m^1^A in Angiogenesis

Also, ALKBH3, the demethylase of m^1^A modification, is named as prostate cancer antigen 1 (PCA-1) (Konishi et al., 2005). The knockdown of ALKBH3 can downregulate expression of VEGF to inhibit the cancer angiogenesis *in vivo* experiment. Also, the overexpressed ALKBH3 is observed in a few human cancers, and its expression is correlated with the TNM stage, such as pancreatic cancer (Yamato et al., 2012).

Compared to the extensively studied m^6^A modification, m^1^A still belongs to the new field of mRNA and ncRNA, and its function has not been widely explored. Therefore, whether m^1^A plays an important role in angiogenesis might be worth exploring.

### Anti-Angiogenic Drugs With RNA Modifications

Many drugs were developed to be anti-angiogenesis in cancer, which is one important strategy in anticancer therapy. In this section, we try to summarize anti-angiogenic drugs which may associate with RNA modifications.

#### Erbitux (Cetuximab)

Cetuximab is an EGFR monoclonal antibody (Chen et al., 2018), which is designed to anti-angiogenesis in CRC, GC, and NHSCC. In the Cetuximab resistant CRC cell, the m^6^A reader hnRNPA2B1 activates the Wnt signaling pathway by stabilizing TCF4 mRNA based on m^6^A-dependent manner (Liu et al., 2022b).

#### Nexavar (Sorafenib)

Sorafenib is a kinase inhibitor which is used in the therapy of HCC, OC, RC and targets for VEGFR, PDGFR, and Raf (Chen et al., 2018). In the physiology condition, the METTL3 is responsible for the methylation site of FOXO3 mRNA to increase its stability. In the Sorafenib-resistant HCC cell, the downregulation of METTL3 affects FOXO3 and its medicated autophagy, which may contribute to the drug resistance (Lin et al., 2020).

#### Sutent (Sunitinib)

Sunitinib is an inhibitor of receptor tyrosine kinase, which provides multi-target therapy in NSCLC, HCC, and PC (Chen et al., 2018). In cells with Sunitinib resistance, METTL14 facilities the stabilization of TRAF1 mRNA to enhance angiogenesis (Chen et al., 2022).

#### Iressa (Gefitinib)

Gefitinib is an EGFR tyrosine kinase inhibitor, which is used in the NSCLC, with a promising therapeutic effect, and is used in EC and NHSCC also (Chen et al., 2018). In a recent study, the NSCLC Gefitin resistance derived from overexpression of VIRMA helps the methylation of HOXA1 to enhance its mRNA stability (Tang et al., 2021b).

#### Tarceva (Erlotinib)

Erlotinib is another kind of EGFR tyrosine kinase inhibitor which is used in the therapy for HCC, PC, and NSCLC patients (Chen et al., 2018). TUSC7, a suppressor of Notch signaling pathway, was inhibited by overexpressing YTHDF2 to contribute to Erlotinib resistance (Li et al., 2021).

#### Nivolumab (anti-PD-1)

PD-1 is an immunosuppressive molecule which acts as a vital role in promoting immune evasion that inhibits antitumor effects. Anti-PD-1/PD-L1 is mainly used for advanced melanoma (Machiraju et al., 2021) and advanced squamous NSCLC treatment (Dantoing et al., 2021). In CRC cells, FTO enables in upregulating the PD-1 expression and thus promoted immune escape (Zhang et al., 2021). Next, ALK-04 (ALKBH5 inhibitor) and suppression of METTL3 and METTL14 increased the efficacy of anti-PD-1 therapy (Li et al., 2020a; Wang et al., 2020c).

## Conclusion and Perspective

In the past the decade, RNA modifications have been proven as an important layer of epigenetics, which regulates gene expression, the cell cycle, and proliferation, fetal development, and progression of disease. Angiogenesis is a vital process in fetal development and associates with tumor metastasis. Here, we reviewed recent studies to summarize the five types of RNA modification-related pathways in angiogenesis. Most studies focused on m^6^A, the most popular mRNA modification, to illustrate its regulatory pathway in physiological and pathological angiogenesis. Other modifications also participate in the regulation of angiogenesis, but they need to be further studied to show the complete regulatory network. In general, aberrant RNA modifications affect the RNA status of certain angiogenic factors or proteins in pathological angiogenesis to promote tumor angiogenesis.

Although the associations between RNA modifications and angiogenesis have been proven in these studies, they mostly focus on a single RNA modification and its corresponding regulators; thus, more aspects of the alteration of angiogenesis could be analyzed further: 1) RNA modification-associated mutations in angiogenesis. The correlations between carcinogenesis and variants of angiogenesis-related genes have been studied in the past few years (Crona et al., 2019; Sullivan et al., 2019). Also, several computational biology projects (Zheng et al., 2018; Song et al., 2020; Chen et al., 2021a; Wang et al., 2021a) suggested that RNA modification-associated mutations may induce diseases or contribute to disease development, and some identified variants were proven further by cohort studies (Meng et al., 2020; Wang et al., 2021b; Ruan et al., 2021). However, no study illustrated how variants affect the RNA modification status to alter angiogenesis. 2) Research on RNA modifications in angiogenesis. The crosstalk between protein modifications (PTMs) and DNA modifications in cancers has been well studied (Cedar and Bergman, 2009; Wu et al., 2019b; Hernandez-Valladares et al., 2019; Li et al., 2020b; Zhang et al., 2020), but RNA modification as an emerging epigenetic layer remains to be discovered. In a recent study for patients with CRC among different RNA modifications, the crosstalk between writers was introduced (Chen et al., 2021b). However, the detailed regulation of crosstalk between different RNA modifications sites in the pathological conditions is still unknown because of technical limitations in current sequencing methods and bioinformatics tools (Chen et al., 2019b; Liu and Chen, 2020; Song et al., 2021b; Xu et al., 2021; Liu et al., 2022c). Previous studies suggested that the different RNA modifications are involved in angiogenesis, and the association among them should be further analyzed. 3) The role of virus infection in angiogenesis. Most RNA modifications, including m^6^A and m^5^C, were proven to be presented on viral RNA, and virus infection has been reported to alter the host epitranscriptome (Courtney, 2021). Also, viral infection is associated with carcinogenesis, which promotes pathological angiogenesis and has been introduced in recent studies (Wu et al., 2022). However, whether infection alters RNA modification to promote angiogenesis is still unclear and should be considered in further studies.

## Glossary

m6A: N6-methyladenosine;
m5C: N5-methylcytosine
m7G: N7-methylguanosine
m1A: N1-methyladenosine
Ψ: pseudopuridine
LC: lung cancer
GC: gastric cancer
BCa: bladder cancer
ICC: intrahepatic cholangiocarcinoma
BC: breast cancer
CRC: colorectal cancer
HCC: hepatocellular carcinoma
CC: colon cancer
CAVMs: brain arteriovenous malformations
UCB: urothelial carcinoma of the bladder
ICD: ischemic cardiovascular disease
PC: pancreatic cancer
ECs: endothelial cells
BMSCs: bone marrow mesenchymal stem cells
AS: atherosclerosis
HUVECs: human umbilical vein endothelial cells
VEGF: vascular endothelial growth factor
FGF: fibroblast growth factor
HDGF: heparin-binding growth factor
MeRIP: methylated RNA immunoprecipitation
miCLIP: m6A individual-nucleotide-resolution cross-linking and immunoprecipitation
METTL3: methyltransferase-like 3
METTL14: methyltransferase-like 14
RBM15: RNA-binding protein 15
VIRMA: virus like m6A methyltransferase associated protein
Zc3h13: zinc finger CCCH domain-containing protein 13
YTHDC: YTH domain-containing
YTHDF: YTH domain-containing family
FTO: fat mass and obesity-related proteins
ALKBH5: alkB homologue 5
IGF2BPs: Insulin-like growth factors
NSUN2: NOP2/Sun RNA methyltransferase family member 2
ALYREF: Aly/REF export factor
YBX1: Y box binding protein 1
MVD: microvessel density
PAD: peripheral arterial disease
PCA-1: prostate cancer antigen 1
